# Global Identification of MicroRNAs and Their Targets in Barley under Salinity Stress

**DOI:** 10.1371/journal.pone.0137990

**Published:** 2015-09-15

**Authors:** Pingchuan Deng, Le Wang, Licao Cui, Kewei Feng, Fuyan Liu, Xianghong Du, Wei Tong, Xiaojun Nie, Wanquan Ji, Song Weining

**Affiliations:** State Key Laboratory of Crop Stress Biology in Arid Areas, College of Agronomy and Yangling Branch of China Wheat Improvement Center, Northwest A&F University, Yangling, Shaanxi, China; East Carolina University, UNITED STATES

## Abstract

Salinity is a major limiting factor for agricultural production worldwide. A better understanding of the mechanisms of salinity stress response will aid efforts to improve plant salt tolerance. In this study, a combination of small RNA and mRNA degradome sequencing was used to identify salinity responsive-miRNAs and their targets in barley. A total of 152 miRNAs belonging to 126 families were identified, of which 44 were found to be salinity responsive with 30 up-regulated and 25 down-regulated respectively. The majority of the salinity-responsive miRNAs were up-regulated at the 8h time point, while down-regulated at the 3h and 27h time points. The targets of these miRNAs were further detected by degradome sequencing coupled with bioinformatics prediction. Finally, qRT-PCR was used to validate the identified miRNA and their targets. Our study systematically investigated the expression profile of miRNA and their targets in barley during salinity stress phase, which can contribute to understanding how miRNAs respond to salinity stress in barley and other cereal crops.

## Introduction

Salinity is one of the most severe environment factors limiting crop yield, which affects about 954 million hectares of land worldwide at present [1]. It’s reported that more than 50% of all arable lands may be salinized because of unreasonable irrigation and climate change by 2050 [2]. Extensive studies have demonstrated that cultivation of salt-tolerant species or varieties is the most economic, efficient and practical approach to alleviate salinity stress in agricultural production [3]. Thus, uncovering the molecular mechanism of salinity response in crops holds the promise for meeting the challenges of food demand increase and global climate change.

MicroRNA (miRNA) is a class of small, non-coding RNAs derived from the processing of longer primary miRNA transcripts, which served as the key players in the gene regulation networks by transcript degradation or translational repression [4]. Since the discovery of miR398 playing an essential role in salt tolerance in *Arabidopsis* [5], a large number of salt-induced miRNAs have been reported in plants up to now, such as miR159, miR160 and so on [5–10]. While some salt related miRNA were identified to show a different expression profile under salinity stress among different plants, such as miR156, miR169 and miR396 [8, 11, 12], which indicated that there might be some species-specific response or tolerance mechanism in miRNA-meditated gene regulation for various plants under salinity stress [13].

As one of the most salt-tolerant cereal crops, barley (*Hordeum vulgare* L.) is the fourth most cultivated crop worldwide and widely grown in arid and semiarid regions [11]. A large number of studies have been conducted to investigate the molecular mechanism and candidate genes responding to salinity stress in barley [12–14]. Some efforts also have been made in recent years to identify miRNA in barley [15–20]. However, the salinity-responsive miRNA in barley have not been well understood up to now. In this study, a combination of small RNA sequencing and mRNA degradome sequencing were used to identify salinity-responsive miRNAs and their corresponding target genes, which will not only provide vital information for the role of miRNAs in salinity stress response, but also shed light on the molecular mechanism of miRNA-mediated genes regulation network under salinity stress in barley and other cereals.

## Materials and Methods

### Plant materials

The barley cultivar Morex, a relative salt tolerant genotype [21] was used in this study. Plant cultures and salinity stress treatment were carried out as previously described [22]. Seeds of barley cv. Morex were sterilized and germinated in vermiculite at 28°C. Then, germinated seeds were transferred to a foam grid with uniform plant density for hydroponic culture. The foam grids were placed on a plastic tanks (length 40 cm, width 30 cm and depth 20 cm, volume 24 L) to suspend the seedling over one-half- strength Hoagland solution (20 L), with the roots immersed in a continuously aerated nutrient solution. The deionized water was added daily to replace the water lost because of transpiration. The experiment was carried out in a growth chamber (ZPG-400B, Dong Tuo, Heilongjiang, China) with 30/20°C(day/night), a relative humidity of 55–65% and the photoperiod of 14h light (6 model)/ 10h dark (0 model). At the three-leaf stage, plants were exposed to salinity by adding NaCl to the growth medium with 25 mmol/L increments every day, until reaching the final concentration of 100 mmol/L NaCl (EC in the range of 14.5–15.5 dSm^-1^). Other plants were grown in culture without NaCl as control. Whole treated plants and counterpart controls were harvested at the 3h, 8h and 27h after reaching the final concentration of 100 mM/L NaCl for downstream experiments. A pool of two replicates was used for small RNA sequencing, whereas three individual plants were used for qRT-PCR validation.

### Small RNA library construction and sequencing

Total RNA was isolated from the above harvested plants using the TRIzol reagent (Invitrogen, USA) according to the manufacturer’s instructions. The RNA quality was examined using gel electrophoresis (28S:18S>1.5) and Bioanalyzer (Agilent2100, RIN≥8.0). The sRNA libraries were constructed following the standard protocol (Illumina, USA). In brief, small RNAs with 16–30 nt were separated from the total RNA by size fractionation. After size selection and purification through agarose gel electrophoresis, the selected fragments were ligated to Solexa adapotors (Illumina, http://www.illumina.com). Then, the ligated RNAs were reverse transcribed and amplified for Illumina sequencing.

The degradome library was constructed using the method as previously described [23]. After salinity stress for 3h, 8h and 27h as described earlier, total RNA from these plants were isolated and pooled with equal amount at each time point. Then, a 5’ RNA adaptor consisting of a Mmel recognition site in 3’ end was ligated into these fragments using RNA ligase. The ligated fragments were reverse transcribed, followed second strand synthesis, Mmel digestion, ligation of a 3’ dsDNA adaptor. Purification of the adaptor-ligated cDNA by agarose gel and then PCR was done to amplify the recovered cDNA for constructing the sequencing library. The high-throughput sequencing was performed on Illumina HiSeq2000 platform according to manufacturer’s instructions at BIOMARKEER Biotech Comp (Beijing, China). Further image analysis and base calling were performed using the Illumina standard pipeline.

### Identification of conserved and novel miRNAs in barley

After poor-quality and adaptor reads filtered, the sequences with length of 16-30nt were retained for further analysis. The small RNAs that were not miRNAs were removed before miRNA analysis by matching to a number of other sources such as chloroplast genome, plant tRNAs, plant repeats and Rfam database. In brief, all trimmed sequences were mapped to the complete barley chloroplast genome without mismatches and then separated into chloroplast reads and non-chloroplast reads (nuclear-derived reads). Then, other classes of nuclear-derived sRNAs were detected by mapping all non-chloroplast reads to Rfam [24] and repeat sequences from TIGR Hordeum Repeats [25], TIGR Oryza Repeats [25], Triticeae repeat sequence database (TREP) [25] and repbase17.11 [26]. Other classes of chloroplast sRNA were analysed by mapping all the chloroplast-derived reads to Hordeum chloroplast gene, Hordeum chloroplast tRNA and Hordeum chloroplast rRNA [27]. All the reads mapping to a given library would be removed and the remaining reads were used to map another library. Known miRNAs were detected by mapping all unannotated reads to barley miRNAs stored in miRBase (v20.0, June 2013) [28] without mismatch. Then, all the reads mapping to barley miRNAs were removed. For putative homologous miRNAs detection, the remaining reads were further mapped (less than 2 mismatches) to other plants miRNA stored at miRBase (v20.0, June 2013). Furthermore, all the unannotated sequences were used to predict novel miRNAs by searching against barley genome sequence [29]. The 100 nt of the genomic sequences flanking each side of the genome-matched sequences were extracted and used to identify novel miRNAs by the Mireap software program [30]. The secondary structure of precursors was further checked using Mfold program [31]. Then, the criteria described by Meyers *et al*. [32] were used to identify the candidate novel miRNAs.

Furthermore, the differentially expressed miRNA were also identified. Firstly, sequences were computed dividing the number of reads of each sample by the total number of reads of each library, normalized per million. Then, the differentially expressed sequence counts were analyzed by the online service IDEG6 (http://telethon.bio.unipd.it/bioinfo/IDEG6_form/). Because of the lack of biological replicates and technical duplicates in sequencing, several relatively stringent criterial were applied to identify differentially expressed miRNA under salinity stress. We considered a fold change of at least 2 (|Log_2_ (treatment/control)| > = 1) as an indication of significant change and P value cut-off (P < 0.00001 simultaneously in Audic Claverie test, Fisher exact test, and Chi-squared 2×2 test with the Bonferroni correction to adjust for pairwise comparison) considered as salinity responsive miRNAs.

### Target prediction for barley miRNAs and degradome sequencing analysis

To identify the potential targets, all the miRNAs identified in this study were used as a query to search against barley DFCI Gene index (HvGI) release 12 using the psRNATarget program with default parameters excepting that the value of Length for complementarity scoring (hspsize) was set to 18 bp [33]. Sequences with total score less than 3.0 points were considered to be miRNA targets.

The raw data of the degradome sequencing were preprocessed to remove low quality reads and clip adapter sequences. Subsequently, only 20–21 nt sequences with high quality scores were collected for further analysis. The barley miRNAs stored at miRBase (Release 20.0) together with the newly identified in this study were used to create a barley miRNA databases. The barley_HighConf_genes_MIPS_23Mar12_CDSSeq from MIPS barley genome database and barley unigene library from DFCI Gene Index (HVGI, version 12, released on 2011) were used to create a barley gene model (including 109,140 barley genes). Bowtie aligner was used to map barley degradome reads to the barley gene model and only the reads perfectly matched to barley transcript sequences were retained. Then, the degradome analysis and the classification of target categories were performed using CleaveLand pipeline (Version 4.3: November 7, 2013) with the barley gene models and all barley miRNA databases as input sets [34].

Furthermore, GO enrichment analysis was performed to understand the function of the potential miRNA target genes [35]. And then, the GO functional analysis of the putative targets was performed by WEGO (http://wego.genomics.org.cn/cgi-bin/wego/index.pl). GO terms with P-value < 0.05 were considered to be significantly enriched.

### Validate the miRNAs and their targets by qRT-PCR

In this study, two methods were used to cDNA synthesis for real-time quantification of miRNAs. One method was based on adding a poly-A tail and a universal adaptor at the 3' position of mature miRNA. In this method, the one step primeScript miRNA cDNA Synthesis kit (Takara Bio Inc., Otsu', Japan) was used to produce the cDNAs, and then amplified with mature miRNA sequence in combination with the universal adaptor as the primer for miRNA expression analysis. The other method was based on a stem-loop RT primer to detect the expression level of miRNAs [36]. The stem-loop reverse transcription reaction was performed in a volume of 20 μl containing 1 uL DNase I-treated RNA,0.5μl dNTP mix (10mM), 0.25μl SuperScript III Reverse Transcriptase (Invitrogen, 200 units/μl), 4 μl 5× First-Strand buffer, 2 μl DTT (0.1 M), 0.1 μl RNase inhibitor-HPRI (Takara, Dalian, China, 40 units/μl), 11.15 μl nuclease-free water and 1μl stem-loop RT primer for each miRNAs (1μM). The cDNA synthesis was conducted in a Bio-Rad C1000 Touch™ Thermal Cycler using a pulse reverse transcription program [36]. Real-time PCR analysis was carried out using SYBR Premix Ex Taq (Takara) on an ABI PRISM 7300 Real-time PCR System using the following condition: 3min at 95°C followed by 40 cycles of 5 s at 95°C and 31 s at 60°C. The amplification specificity was monitored by melting curve after PCR. The qRT-PCR was performed in three replications. The primers are listed in S1 Table and the housekeeping gene 18s RNA was used as reference. The 2^-△△Ct^ method was used to analyze the relative changes in miRNA expression where Δ*C*_T_ = (*C*_TmiRNA_ − *C*_T18S rRNA_) and ΔΔ*C*_T_ = (Δ*C*_T treatment_ -Δ*C*_T control_) [37].

## Results and Discussion

### Deep sequencing of small RNAs in barley under salinity stress

**Fig 1:**
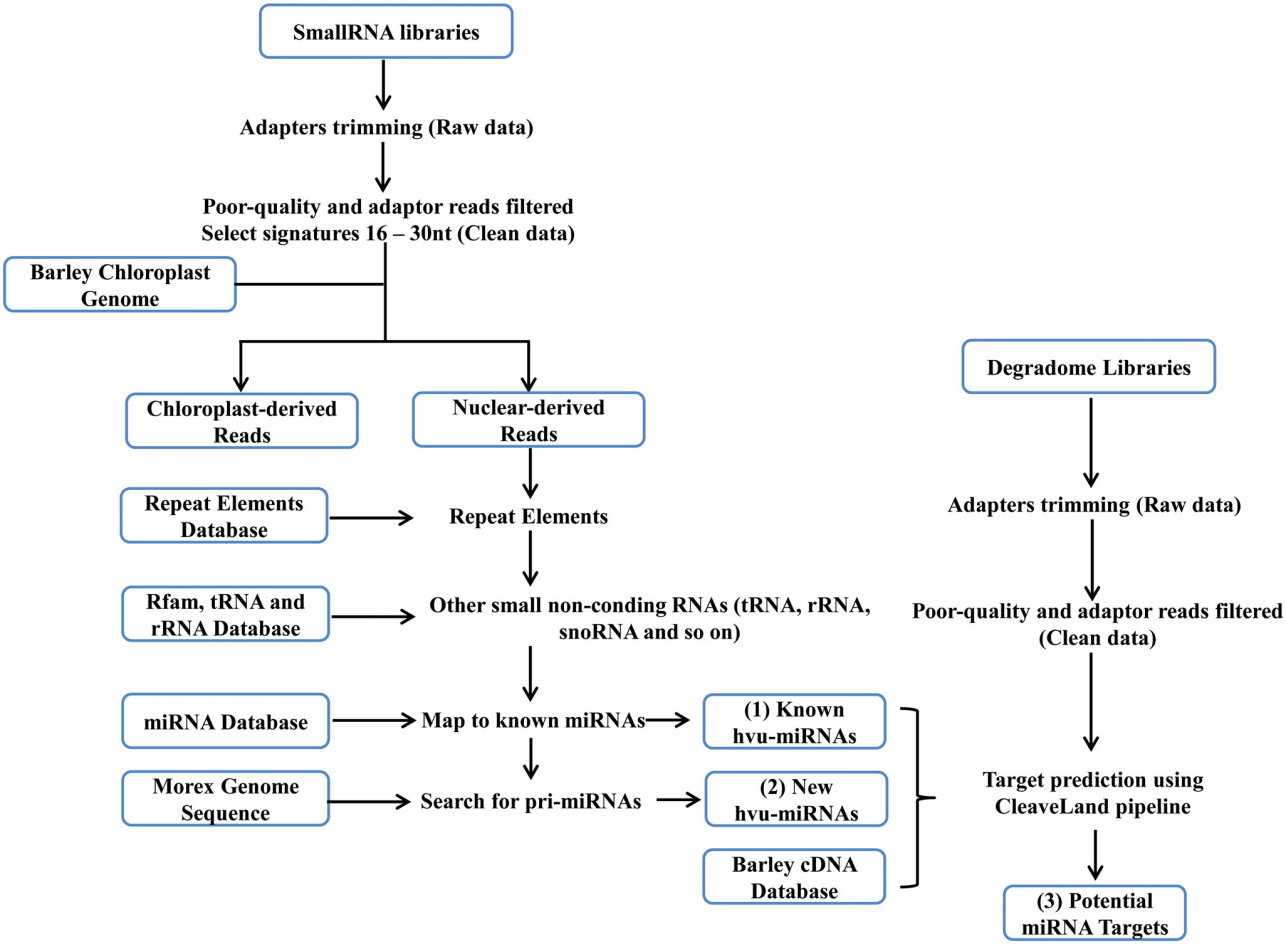
Flow diagram of smallRNA and degradome library analysis. Note: Six smRNA libraries and one mRNA degradome library were made from whole barley seedling under salinity stress and counterpart control (3h, 8h and 27h). Repeat elements database included repeat sequences from TIGR Hordeum Repeats, TIGR Oryza Repeats, Triticeae repeat sequence database (TREP) and repbase17.11. The miRNAs identified from the analysis of the small libraries (including (1) known miRNA and (2) new miRNAs) and barley miRNAs stored at miRBase (v20.0, June 2013) were used in combination with the degradome libraries to find potential miRNA regulated genes.

As shown by previous studies [38], there are three key phases of plant response to salinity stress, including reducing growth rate phase (3h), growth rate recovery phase (8h) and ion-specific responses (27h). To elucidate the miRNA-mediate mechanisms of salinity stress tolerance in barley, six RNA libraries (salinity treated samples and their corresponding control at 3h, 8h and 27h) were generated and sequenced using Illumina technology. An overview of our analysis was presented in Fig 1. An average of 22.34 million sequence tags were obtained, with the largest number of 27.83 million for 27h treatment and the lowest number of 18.75 million for 27h control (S2 Table) (high-throughput sequencing data were deposited into the NCBI database with accession no. SRX1176317). After quality filtering, a range from 8.91 million (27h, control) to 14.33 million (27h, treatment) clean reads were remained, of which approximately 65.2% of these clean reads could be mapped to the genome sequence of barley cv. Morex and 5.8% was Exon/Intron tags [29].

**Fig 2:**
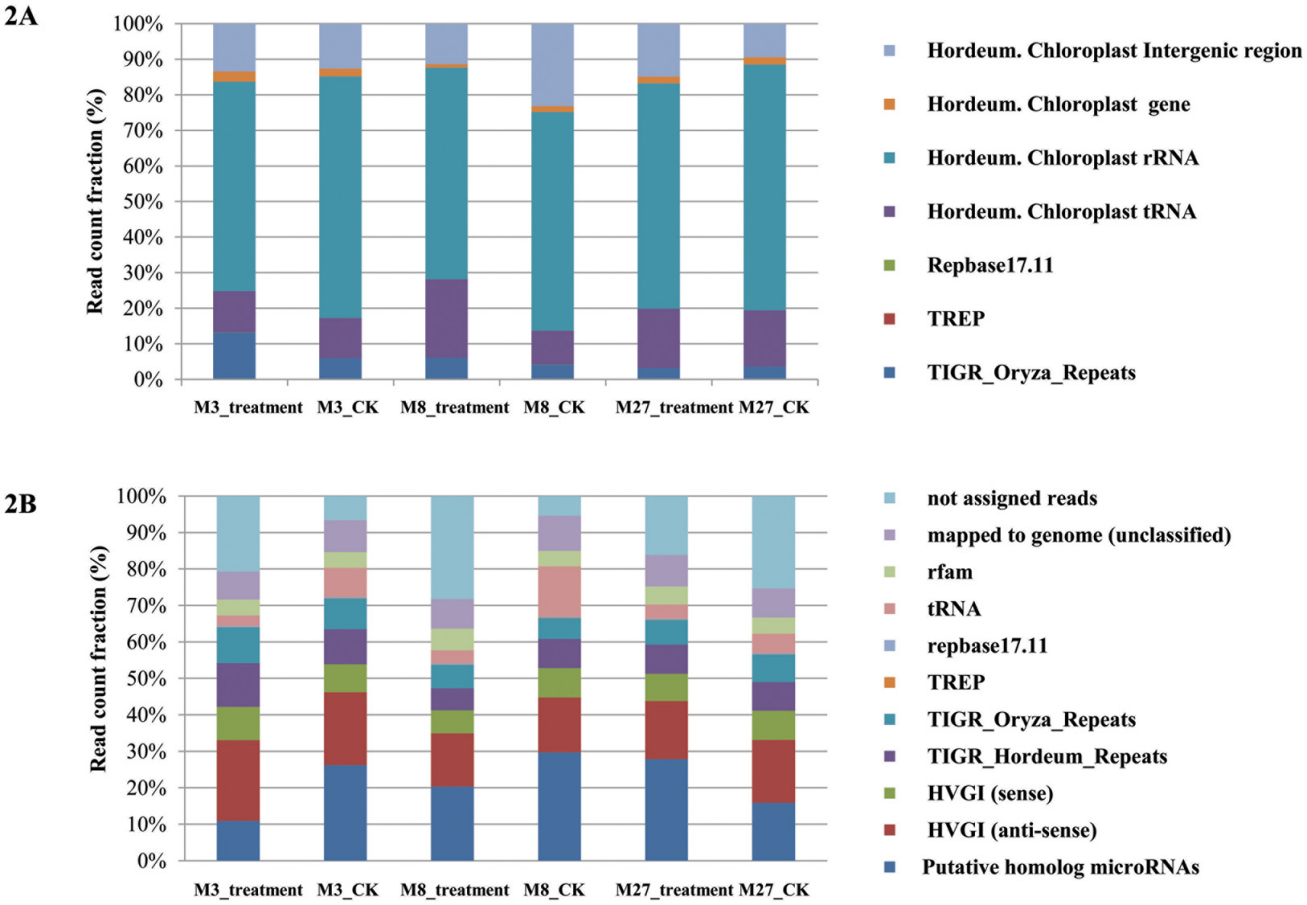
Distribution of different classes of sRNAs derived from barley chloroplast genome (A) and nuclear genome (B).

Firstly, the chloroplast-derived sRNAs (csRNAs) were identified by mapping all trimmed sequences to the complete barley chloroplast genome (GI: 118430366) without mismatches. Averages of 2,335,565 csRNAs were obtained, of which 82, 322 were unique accounting for 19.94% of the total sRNAs (S2 Table and Fig 2A). For total reads, the csRNAs derived from rRNA were the most abundant and these from mRNA were the least prevalent, while for unique reads, csRNAs derived from tRNA were the least prevalent. These results were consistent with that of Chinese cabbage under heat stress [39]. At the same time, 21.49% of the unique csRNA reads and 39.08% of total csRNA reads could be mapped to repeat elements. Among them, 38.99% of the total csRNA were mapped to TIGR Oryza Repeats, 0.01% and 0.08% mapping to TREP and Repbase17.11, respectively.

In contrast to sRNAs derived from the chloroplast genome, nuclear-derived sRNAs were highly redundant under both salinity treatment and control,with an average of 11,715,135 (80.06%) total reads and 2,232,374 (96.31%) unique reads (S2 Table and Fig 2B). To classify the function of these small RNA, all the nuclear-derived sRNAs were further grouped by mapped to miRbase (Version 20), TIGR repeat database, TREP repeat database, RepBase, all plants tRNA databases and Rfam. We observed that an average of 82.94% reads could be assigned to the above databases. Among them, 22.09% of them were mapped to plant miRNA, 16.60% to repetitive elements (8.71% for TIGR Hordeum Repeats, 7.53% for TIGR Oryza Repeats, 0.07% for TREP and 0.29% for repbase17.11, respectively), 6.03% to tRNA, 4.70% to ncRNAs from the Rfam database, and 8.49% to un-annotated genomic regions. The antisense strands of barley genes (from the HVGI database), accounting for 17.58%, occupy the highest proportion among all the mapped sRNAs excepting miRNA.

### Identification of conserved and novel miRNAs in barley

Using the methods as described in the Materials and Methods, a total of 28 barley miRNAs from 20 miRNA families were detected (S3 Table). All the barley miRNAs except Hvu-miR5050 were concurrently detected under salinity stress and normal condition. Hvu-miR5050 was just detected at the 8h and 27h under salinity stress, although with very low reads. In addition, we have identified both mature and star sequences for two barley miRNAs (hvu-miR168 and hvu-miR171). Furthermore, hvu-miR171* showed a higher expression than hvu-miR171 under both salinity stress and normal condition.

After excluding the barley miRNA-mapping reads, the remaining ones were further mapped (less than 2 mismatches) to other plant miRNA in miRBase. A total of 114 putative homologous miRNAs from 99 miRNA families were identified (S3 Table). Among them, 61.97% of miRNAs showed a bias toward 5 uridine or 3 guanosine terminal residues, which was similar to the characteristic of wheat miRNAs [40]. The length of mature miRNAs ranged from 18nt to 24nt. The majority of the small RNA were 21nt in size, occupying for 59.86% of the total conserved miRNAs, followed by 22nt (11.97%), 24nt (10.56%), 20nt (9.86%) and 23nt (3.52%), while the proportion of 19nt and 18nt- sRNA were quite small, only 2.82% and 1.41%, respectively (S1 Fig). Furthermore, we found a high abundance for miRNA*sequences of 14 miRNAs (S2 Fig) [28]. Additionally, the star strand of three miRNAs (bdi-miR159b*, bdi-miR166b*, and ata-miR5168*) showed a higher abundance than their corresponding mature sequence (S2A Fig), while other four (ata-miR156d*, osa-miR160f*, ata-miR167b* and ata-miR396e*) showed a less abundance (S2B Fig). Two miRNAs showed a similar expression of the mature and star strands (ata-miR172b* and ata-miR396e) (S2C Fig). The remaining five miRNAs (tae-miR9662a, zma-miR395b, tae-miR1130b, bdi-miR827 and bdi-miR408) were just detected with the star strands (S2D Fig).

**Fig 3:**
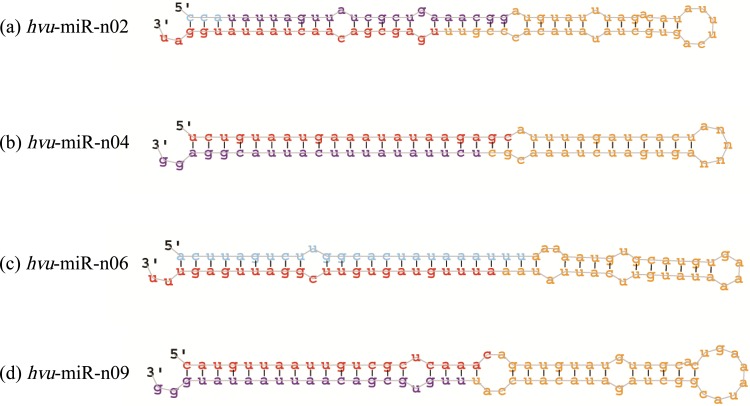
Pre-miRNA stem-loop structures for partial novel miRNAs identified in this study. Note: Predicted mature miRNA sequences are shown in red.

Furthermore, some novel miRNAs were identified. A total of 10 small RNAs can meet the criteria as describing in Materials and Methods, which were considered as putative novel miRNAs (S4 Table). The length of these novel miRNAs ranged from 18 nt to 24 nt, with 21 nt representing the most abundance class (8/10, 80%). Precursors were also identified among the barley genome sequences (S4 Table and Fig 3). The size of these miRNA precursors ranged from 70 nt to 85 nt with the average of 77 nt. Homologous sequences of miR-n02, miR-n04, miR-n06, miR-n08 and miR-n09 were also found in wheat by aligning the candidate precursor against the survey sequence of *T*. *aestivum* (https://urgi.versailles.inra.fr/blast/blast.php), while the other five miRNAs were only found in barley. Significantly, the majority of the novel miRNA was detected in all six samples with various expression levels. The obtained sequence count data for these miRNAs in each of the libraries suggested that the depth of sequencing could be sufficient to support estimation of their expression levels. However, some specific-expression of the novel miRNAs was also found, with miR-n07 only detected at 3h salinity treatment, while miR-n09 and miR-n10 in all six libraries except 3h and 8h salinity treatment (S4 Table). These specific-expression miRNA may involve n the regulation network of barley response to salinity stress and play a vital role in regulating gene expression and metabolism process during salinity treatment.

**Fig 4:**
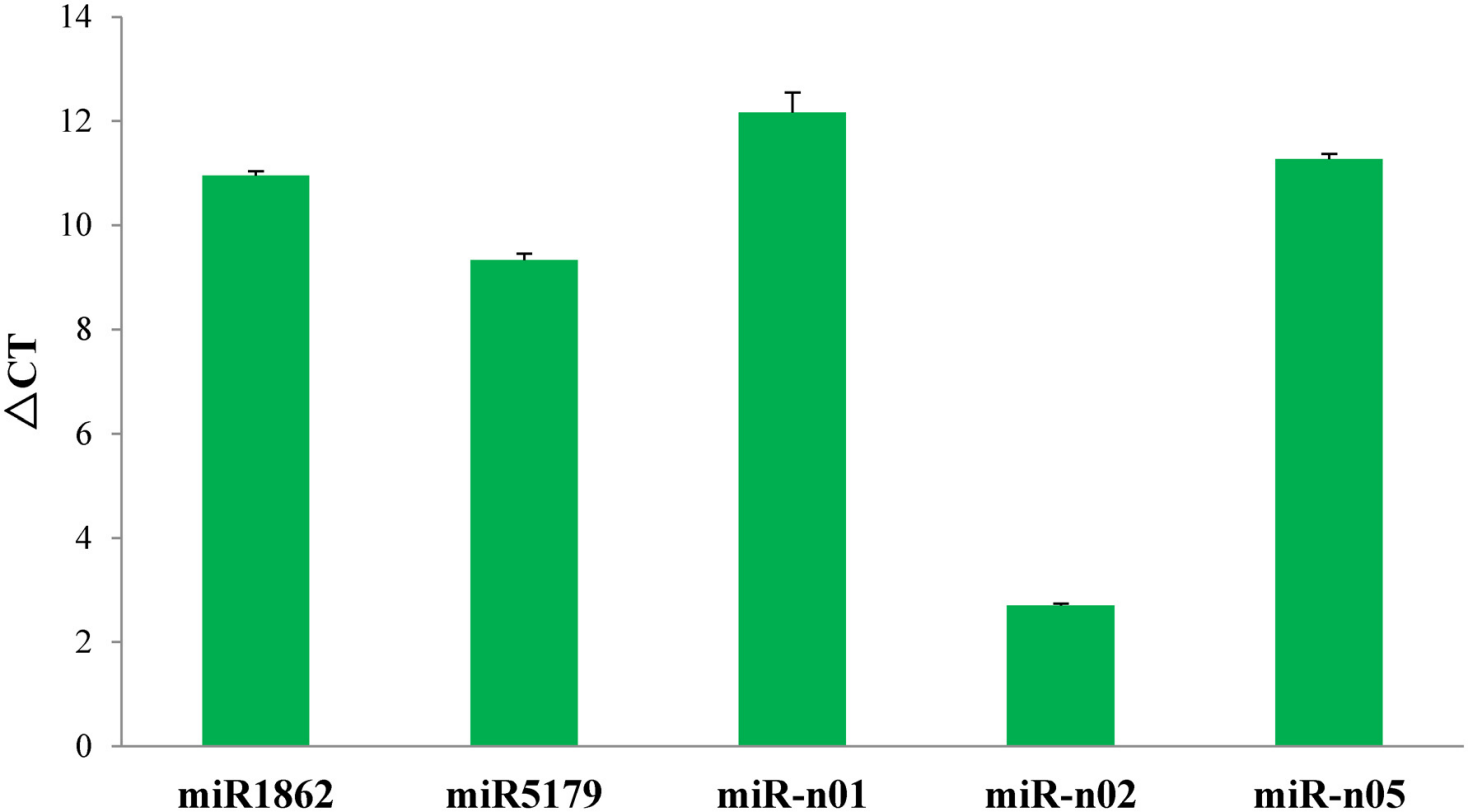
Quantitative real-time PCR analysis of parts miRNA identified in barley. Note: The amount of expression was normalized to the level of 16s RNA.

To validate these newly identified miRNAs, 5 miRNAs were selected randomly to detect expression level in seedling with 3h salinity treatment where the expected product was recovered for all the selected miRNAs by qRT-PCR analysis. Results showed that all the five miRNAs were detected and miR-n02 and miR5179 showed a higher expression, while the miR1862, miR-n01 and miR-n05 showed a lower expression (Fig 4). The results of qRT-PCR analysis also provided the evidence for the actual existence of these novel miRNAs.

### Target genes characterization for the identified miRNAs by bioinformatics prediction and degradome sequencing

To understand the biological function of these identified miRNAs, their targets were first predicted through bioinformatics analysis. Totally, 1589 candidate target genes were identified for 152 miRNA using psRNATarget program (2020 target genes for 142 conserved miRNA and 456 target genes for 10 novel miRNAs, respectively), with the average of 13 target genes per miRNA (S5 and S6 Tables). In addition, the majority of the target genes (67.92%) were regulated by transcript degradation, while the remaining was regulated by translational repression. A total of 5 miRNAs were found to regulate target genes at two positions (S5 Table). Furthermore, 132 genes targeted by more than one miRNA were also found(S5 Table). These phenomena were also observed in wheat, suggesting that similar miRNA regulation systems and evolutionary events may exist in both wheat and barley [40, 41].

Furthermore, degardome sequencing was used to identify the target genes of these barley miRNAs. It’s well known that the majority of the plant miRNAs characterized to date interacts with their target genes by miRNA directed cleavage at the tenth nucleotide of complementarity, resulting in 3’ target fragments had a 5’ monophosphate and a 3’ poly-A tail, which makes it possible to identify target genes by degradome sequencing. We pooled the RNA samples from 3h, 8h and 27h to construct the library for degradome sequencing. Therefore, a total of 5,438,888 raw reads were produced (S3 Fig). After quality filtering, 4,928,699 unique reads were obtained and 93.66% of these reads were 20 or 21 nt in size. Among them, 86.68% could be mapped to the barley reference sequence. The computationally predicted gene and cDNA transcripts of barley were also used as the mapping template sequences of the degradome sequencing reads and found that 83.09% of them could be perfectly mapped to the barley gene models, representing 22,972 annotated barley genes. When a mismatches threshold < = 2 was applied, nearly 90% (4,389,591/4,928,699) of unique reads could match to the barley genes and represent 85.24% (93,029/109,140) of the barley gene.

**Fig 5:**
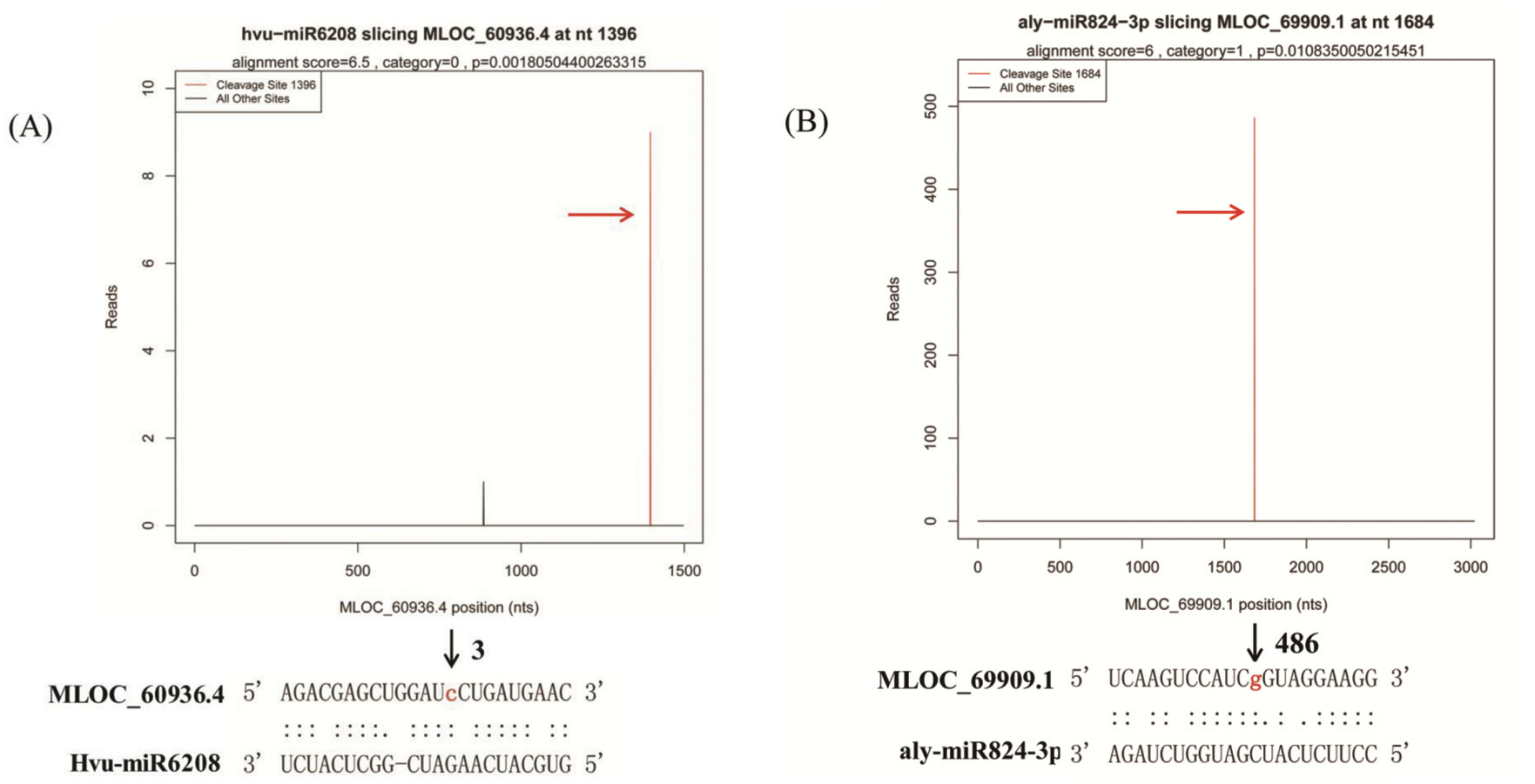
Examples of Target plots (T-plots) of identified miRNA targets using degradome sequencing. Note: The T-plots show the distribution of the degradome tags along the full length of the target mRNA sequence. Red arrows indicate signatures consistent with miRNA-directed cleavage. mRNA:miRNA alignments along with the detected cleavage frequencies (absolute numbers) are shown above the black arrow. The red colored lowercased nucleotide on the target transcript from 3’ end indicates the cleavage site detected in the degradome library.

Furthermore, using the method of CleaveLand pipeline as described in Material and methods [34], a total of 65 targets were identified for 39 different miRNAs, including 37 conserved miRNAs and 2 novel miRNAs (S7 Table). The cleaved target transcripts were categorized into five classes (categories 0, 1, 2, 3 and 4) based on the abundance of degradome tags indicative of miRNA-mediated cleavage [42]. Among the identified targets, category 2 was the most abundant category occupying for 29.23%, followed by category 3 (27.69%), category 1 (16.92%), category 0 (15.38%) and 4 (10.77%), which was consistent with the results of previous study [42]. The miRNAs were found to be able to target various numbers of genes, ranging from 1 to 6, of which miR951 had the highest number of targets, targeting 6 unique genes (S7 Table). Examples of the ‘target plots’ (T-plots) of targets for identified barley miRNAs were shown in Fig 5.

### Expression analysis of miRNA and their targets by qRT-PCR

**Fig 6:**
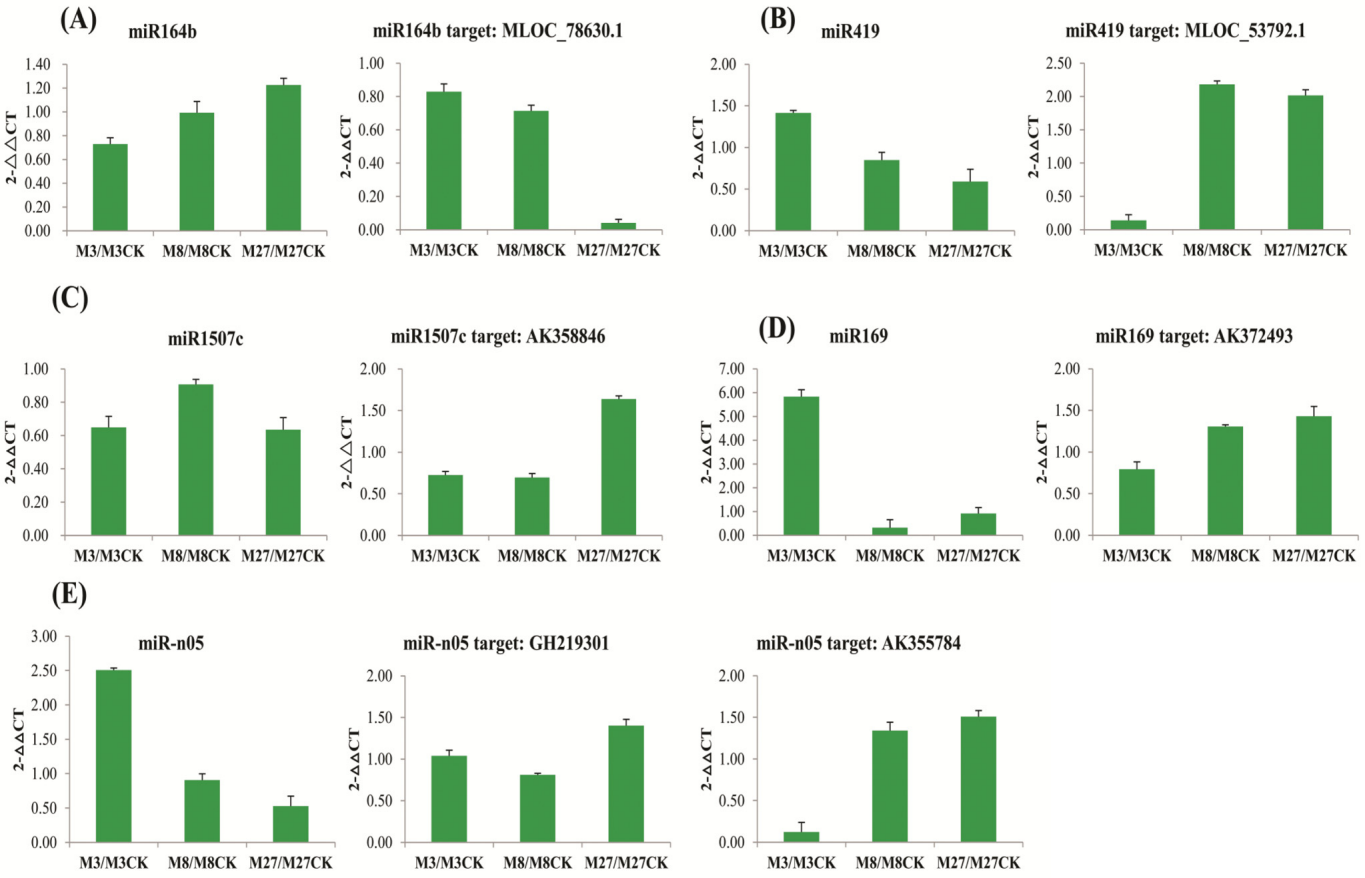
Quantitative real-time PCR analysis of miRNAs and their targets under salinity stress and normal condition. Note: The amount of expression was normalized to the level of 16s RNA. The normalized miRNA levels in control were arbitrarily set to 1.

To validate the targets of the miRNAs, 5 miRNAs and their target genes which were verified by the degradome sequencing, were selected to further confirm by qRT-PCR (Fig 6). The qRT-PCR results showed that four out of these targets have a reverse expression level compared to their counterpart miRNAs. miR164b was down-regulated at 3h and 8h, but up-regulated at 27h, while its expression level showed a gradual increase following the time of salinity stress (Fig 6A). The target gene of miR164b (Oxygen-evolving enhancer protein, MLOC_78630.1) showed a gradual decrease following the time of salinity stress in addition to reverse down or up- regulation patterns of miR164b at the same time points of salinity stress. In contrast to miR164, miR419 showed a gradual decrease whereas its target gene probable cytokinin riboside 5 (MLOC_53792.1) displayed a gradual increase, following the time of salinity stress (Fig 6B). miR169 was up-regulated at the 3h time point, while down-regulated at the 8h and 27 time points. And its target gene polyubiquitin 10 (AK372493) displayed a contrast expression patterns (Fig 6D). miR1507 appeared down-regulated at all the three time points, while its target gene translation initiation factor eIF-2B subunit alpha did not show a strict reverse expression pattern compared with that of miRNA (Fig 6C). This target gene was down-regulated at 3h and 8h, but up-regulated at 27h. It might indicate that there could be some time lag for certain miRNAs to initiate regulation of their target genes. Similar results were also found with miR-n05 and its target gene enolase (GH219301). However, another target gene (B-cell receptor-associated protein, AK355784) of miR-n05 displayed a contrast expression pattern with their miRNAs (Fig 6E). It might indicate that a delayed regulation pattern to initiate regulation of their target genes may be a method for miRNA mediated multiple target genes regulation under salinity stress.

### Analysis of salinity responsive miRNAs in barley

Through comparing the miRNA profiles between salinity treated samples and their control, the salinity responsive miRNAs were further identified as described in Materials and Methods. Among the 28 barley miRNAs, 6 were salinity responsive miRNAs (Table 1). Three miRNAs (hvu-miR168-5p, hvu-miR5048a and hvu-miR444b) were down-regulated while two other miRNAs (hvu-miR171-5p and hvu-miR6213) were up-regulated under salinity stress. The left miRNA (Hvu-miR444a) significantly expressed at all the three time points, which was down-regulated at 8h and 27h but up-regulated at 3h. All of the salinity responsive novel miRNAs except hvu-miR-n01 were up-regulated under salinity stress at the 3h and 8h time points, while all of them except hvu-miR-n05 were down-regulated at the 27h time point (Table 1 and S8 Table). For putative homologous miRNAs, a total of 26 miRNAs and six miRNA* were significant differential regulation at one or more of the three time points.

**Table 1:** List of differentially expressed miRNAs identified in barley under salinity stress.

Proposed miRBase name	miRNA sequence	log2(M3h_treatment/M3h_control)	log2(M8h_treatment /M8h_control)	log2(M27h_treatment/M27h_control)
**Barley miRNA**				
hvu-miR168-5p	aagccaacugauacuaauugg	-2.27	-1.1	0.47
hvu-miR171-5p	uauuaguuaucgcugaaacgg	-0.63	-0.01	2.79
hvu-miR444a	ucuuauauuucggaauggagg	1.64	-3.32	-1.32
hvu-miR444b	gcuuuguuccuuggauuucca	-1.49	-0.2	-0.6
hvu-miR5048a	auuuguaguguucggauugaguuu	-2.7	-2	0.82
hvu-miR6213	acagauugcuacagacugguc	2.83	0.04	-0.24
**Barley novel miRNA**				
hvu-miR-n0	aagccaacugauacuaauugg	-1.22	-2.6	-3.25
hvu-miR-n02	gauggcaggugacgguguugacgc	1.89	2.16	-0.45
hvu-miR-n03	agcacuggagguccgaaacca	0.63	2.06	0.35
hvu-miR-n05	gcuuuguuccuuggauuucca	0.98	-1.25	-3.56
hvu-miR-n06	cggccaagagaggaguccucccu	-0.03	1.44	-2.06
**Putative homologous miRNAs**				
ata-miR167b-3p	aggucaugcuggaguuucauc	-0.99	1.5	0.35
ata-miR167b-5p	ugaagcugccagcaugaucuga	-1.88	3.13	0.76
ata-miR172b-3p	agaaucuugaugaugcugcau	-1.93	-1.93	1.22
ata-miR396e-5p	uuccacagcuuucuugaacug	-1.96	-1.12	0.23
bdi-miR1135	uuucgacaaguaauuccgaccgga	-1.11	-1.81	2.22
bdi-miR159b-3p.1	uuuggauugaagggagcucug	-1.48	-0.42	-2.97
bdi-miR159b-5p.1	gagcuccuaucauuccaauga	1.27	0.55	-1.77
bdi-miR164b	uggagaagcagggcacgugca	-2.11	-0.13	3.16
bdi-miR1878	acuuagucugaacacuauaaaaaa	-3.06	0.1	2.99
bdi-miR319a	ugagggagcuuucuucugucc	-5.51	1.26	0.54
bdi-miR393b	uccaaagggaucgcauugauc	-2.27	5.03	-6.63
bdi-miR408-3p	cugcacugccucuucccuggc	-1.63	0.29	-2.67
bdi-miR5176	uaugccaugucgucacauauc	-6.42	1.64	-0.52
bdi-miR5179	uuuugcucaagaccgcgcaac	-0.97	1.48	0.48
bdi-miR5200	uguagauacucucuaaggcuu	-1.72	-0.02	-0.41
bdi-miR827-3p	uuagaugaccaucagcaaaca	-0.36	-1.11	0.54
gma-miR1507c	gagguguuugggaugagagaa	-1.63	-0.83	-1.34
gma-miR5368	ggacagucucagguagaca	1.16	0.9	-1.49
osa-miR169a	cagccaaggaugacuugccga	-4.27	-5.36	4.96
osa-miR319b	uuggacugaagggugcuccc	-0.9	6.39	4.81
osa-miR419	ugaugaaugcugacgauguug	4.85	3.1	-2.49
osa-miR5071	ucaagcaucauaucguggaca	-1.44	-0.58	-1.37
osa-miR5072	cgauuccccagcggagucgcca	1.71	0.58	-1.57
osa-miR5144	uucuugugcugcugaagagac	-1.47	4.47	-2.21
osa-miR5485	ugacaacugguagcagagcaa	3.59	1.87	-0.73
osa-miR5529	guuucauccauggacaccgca	2.57	1.61	-1.04
osa-miR5538	acugaacucaaucacuugcugc	1.77	1.2	-1.3
osa-miR818c	aaucccuuauauuaugggacgg	3.23	1.93	1.34
ppa-miR894	cguuucacgucggguucacc	1.5	0.59	-0.88
ptr-miR169s	ucagccaaggaugacuugccg	-1.26	3.42	-4.6
tae-miR1122	uagauacauccguaucuaga	0.15	5.2	-0.33
tae-miR9662a-3p	uugaacaucccagagccaccg	-1.33	-0.61	0.22
zma-miR395b-3p	gugaaguguuugggggaacuc	-3.09	-0.17	-2

Note: The fold change at the three time points sampled is expressed as Log2 (treatment/control). Fold changes more than 1.0-fold are significant changes and marked in bold.

Taken together, a total of 44 miRNAs belonging to 39 miRNA families were identified as salinity responsive, of which 6 were barley conserved miRNA and 5 were newly identified in this study (Table 1 and S8 Table). Among them, 29 miRNAs from 27 miRNA families were up-regulated while 27 miRNAs from 25 miRNA families were down-regulated. Previous study has demonstrated that under salinity stress, the majority of the barley functional genes were down-regulated at 8h but up-regulated at the 3h and 27h [22]. In this study, the majority of the salinity responsive miRNAs (57.14%) were up-regulated at the 8h while down-regulated at the 3h and 27h time points (62.50% and 65.00% respectively, Table 2). The contrast expression patterns of the salinity responsive miRNAs and functional genes, suggest that the potential roles of these salt-responsive miRNA playing in regulating the gene expression under salinity stress in barley.

**Table 2:** The overview of miRNAs highly responsive to salt stress at the three time points.

Time after sanility stress	Total miRNAs	Up-regulated miRNAs	Down-regulated miRNAs
3h	32	12 (37.50%)	20 (62.50%)
8h	21	11 (52.38%)	10 (47.62%)
27h	20	6 (30.00%)	14 (70.00%)

### Functional Enrichment for the identified salinity responsive miRNA Target genes

To gain a better understanding of the functional roles of the targets of salinity responsive miRNA, we further performed the functional enrichment of these targets by Gene Ontology (GO) analysis. The predicted targets were sorted into 616 GO terms. Among them, 396 were Biological Process-related pathways and 155 were Molecular Function-related pathways (Table 3). In Biological Process, we found the frequency of biosynthetic process, metabolic process, defense response and response to stimulus-related (such as response to stimulus, response to stress and response to chemical stimulus) GO terms were increased under salinity stress, whereas the growth and organ growth-related GO terms was decreased. Interesting, nitrogen compound metabolic process GO term was also found to be increased under salinity stress, suggesting that high metabolic process for plants to cope with salinity stress would require re-adjustment of nitrogen (N) homeostasis. In Molecular Function, the frequency of binding, nucleotide binding, catalytic activity and hydrolase activity were increased under salinity stress but protein binding were decreased. The differences of these GO terms for barley under salinity stress seemed consistent with the observations of functional genes reported by Walia *et al*. (2006).

**Table 3:** Functional categories of genes targeted by salt responsible miRNAs in barley.

GO ID	Term	Observed	Expected	P value
**Biological Process (Total: 15)**				
GO:0008152	metabolic process	156 (65.5%)	6050 (32.8%)	0
GO:0006807	nitrogen compound metabolic process	81 (34.0%)	2321 (12.6%)	0
GO:0009058	biosynthetic process	59 (24.8%)	3281 (17.8%)	0.005
GO:0043170	macromolecule metabolic process	95 (39.9%)	3963 (21.5%)	0
GO:0044237	cellular metabolic process	129 (54.2%)	5309 (28.8%)	0
GO:0044238	primary metabolic process	121 (50.8%)	4907 (26.6%)	0
GO:0009987	cellular process	147 (61.8%)	6990 (37.9%)	0
GO:0044237	cellular metabolic process	129 (54.2%)	5309 (28.8%)	0
GO:0003006	reproductive developmental process	15 (6.3%)	707 (3.8%)	0.049
GO:0043473	pigmentation	50 (21.0%)	2530 (13.7%)	0.001
GO:0019222	regulation of metabolic process	36 (15.1%)	1461 (7.9%)	0
GO:0050896	response to stimulus	59 (24.8%)	3146 (17.0%)	0.002
GO:0065007	biological regulation	53 (22.3%)	2776 (15.0%)	0.002
GO:0040007	growth	7 (2.9%)	1578 (8.6%)	0.002
GO:0044085	cellular component biogenesis	8 (3.4%)	2155 (11.7%)	0
**Molecular Function (Total: 9)**				
GO:0003824	catalytic activity	130 (54.6%)	4672 (25.3%)	0
GO:0016491	oxidoreductase activity	24 (10.1%)	936 (5.1%)	0.001
GO:0016740	transferase activity	51 (21.4%)	1265 (6.9%)	0
GO:0016787	hydrolase activity	43 (18.1%)	1628 (8.8%)	0
GO:0005488	binding	150 (63.0%)	5600 (30.3%)	0
GO:0000166	nucleotide binding	81 (34.0%)	967 (5.2%)	0
GO:0001882	nucleoside binding	66 (27.7%)	612 (3.3%)	0
GO:0043167	ion binding	36 (15.1%)	700 (3.8%)	0
GO:0005515	protein binding	10 (4.2%)	2773 (15.0%)	0

Note: Observed, numbers of genes observed in this study; Expected, numbers of genes in this same category in the GO enrichment analysis program.

### Comparative analysis of the salinity induced miRNAs at the three time points

**Fig 7:**
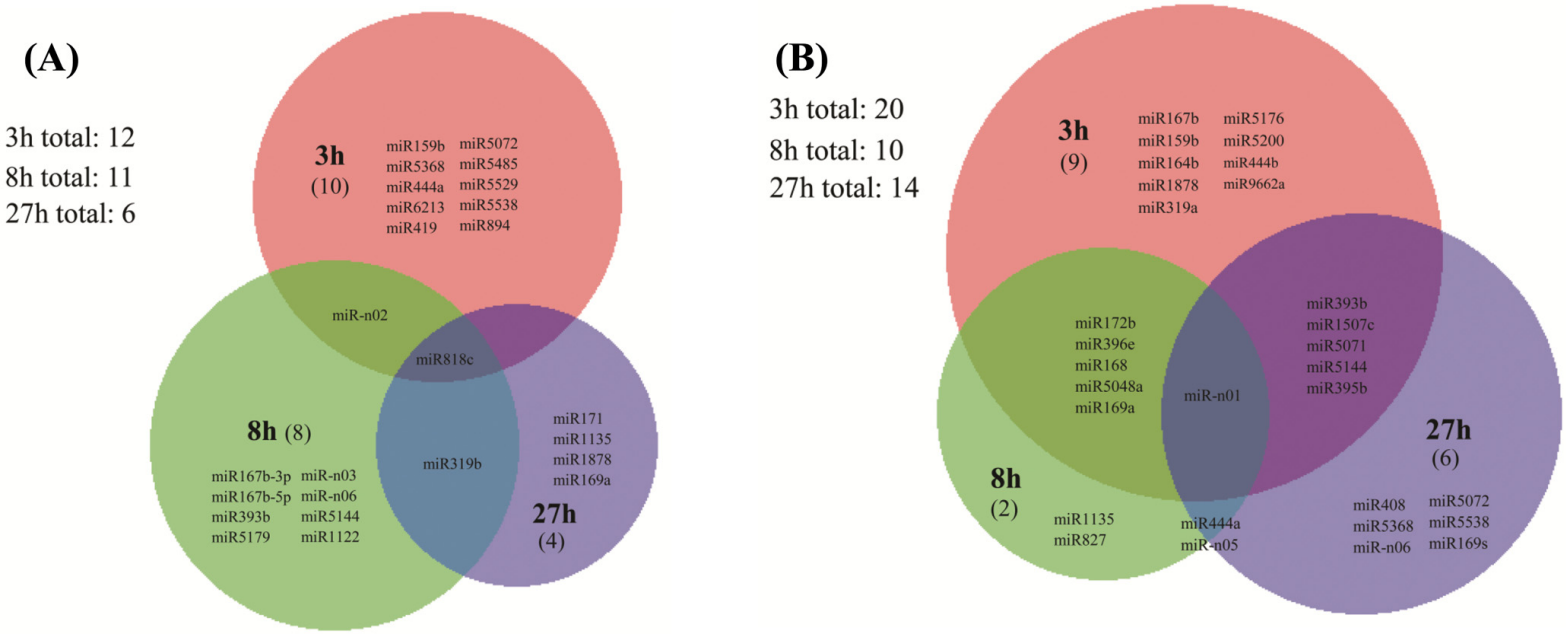
Venn diagram of salt responsive miRNAs at 3h, 8h and 27h. Note: (A) Up-regulated miRNAs in response to salinity stress. (B) Overlap among the down-regulated miRNAs.

Comparative analysis of these salinity responsive miRNAs at the three key phases was further performed to identify the main candidate miRNAs in salinity response of barley. Most of the up-regulated or down-regulated miRNAs showed a time specific expression pattern. For up-regulated miRNAs, 10, 8 or 4 of them were found to be expressed specifically at the 3h, 8h and 27h time point, respectively. And 2 of these miRNAs were shared by two time points (1 between the 3h and 8h, 1 between the 8h and 27h), with only one miRNA (osa-miR818c) shared by all the three time points (Fig 7A). Among the down-regulated miRNAs, there were just one miRNAs (hvu-miR-n01) shared by three time points, while 12 miRNAs were shared by two time points (Fig 7B). Finally, 9, 2 and 6 down-regulated miRNAs were specifically expressed at the 3h, 8h and 27h time points, respectively.

In the present study, a number of miRNAs with targets involved in modulating leaf and root development displayed significant differential expression under salinity stress. Among them, miR164b was down-regulated while miR160f was up-regulated. NAC-domain proteins, the targets of miR164b, have been reported to be salinity induced in many species [7, 43]. Previous studies showed that NAC1 might be an early auxin-responsive gene and positively regulate the lateral root development [44]. The increasing accumulation of NAC1 transcripts resulting from the down-regulated miR164b would promote lateral root development at the 3h time point. At the same time, miR172b was down-regulated while its target gene auxin response factor (ARF) was up-regulated at the 3h and 8h time points. ARF regulates various important processes in plants growth, development and responses to environmental stress [45, 46]. The down-regulation of miR172b would increase the expression of ARF, thus promoting auxin response and enhance leaf development. Moreover, it has been reported that a decrease in endogenous cytokinins (CKs) levels could enhance salinity stress tolerance [47]. In this study, a cytokinin biosynthesis related gene (cytokinin riboside 5’-monophosphate phosphoribohydrolase, MLOC_53792.1) was found down-regulated at the 3h time point, while up-regulated at the 8h and 27h as a result of regulation by miR419 (S7 Table and Fig 6),indicating that miR419 might play a role in regulating barley growth under salinity stress. Our results indicate that these miRNAs might play a role in modulating barley morphological development to cope with salinity stress.

Salinity damage might be the result of excess apoplastic ion concentrations or ion toxicity effects on metabolic processes in the symplast including inhibited most enzymes activity, damaged membranes and macromolecules [38]. Heat-shock proteins (Hsps), acting as molecular chaperones and maintaining the stabilization of proteins and membranes, were one of the major types of specific stress-induced proteins that accumulated upon water, salinity and extreme temperature stress [38, 48]. miR396e was predicted to target Hsp80, responsible for both heat-shock and developmental regulation [49]. In our study, miR396e was down-regulated at the 3h and 8h time points of salinity stress, likely resulting in the upturn of Hsp80 transcripts and consequently maintaining the stabilization of proteins and membranes. On the other hand, plant also degraded some mis-folded proteins and those proteins no longer needed by the cell to cope with stress. Heat shock protein HslVU was a proteasome-like degradation complex and responsible for 70 to 80% of the protein degradation *in vivo* [50]. miR5175a was down-regulated at 8h time point and predicted to target HslVU, which might assure the expression of HslVU degradation of some mis-folded proteins in response to stress.

Recently, wheat enolase was identified as a non-toxic cryopreservation agent and it could protect cells and tissues against oxidative stress [51]. In our study, miR-n05 was detected to target enolase (GH219301) by degradome sequencing (S7 Table). qRT-PCR also showed that the enolase was up-regulated at the 27h time point resulting from the down-regulation of miR-n05 (Fig 6E), indicating that miR-n05 might be involved in defense against the oxidative effect generation during salinity stress.

There also may be a negative feedback regulation in the miRNA pathway for barley under salinity stress. In plants such as Arabidopsis [5], maize [52] and cotton [53], miR168 was detected to target AGO1 (ARGONAUTE 1)) gene and involved in miRNAs biogenesis and function. AGO1 genes (Hv.19452 and Hv.26206) were also found targeted by miR168 in barley [54]. In this study, miR168 was found to be a salinity responsive miRNA and down-regulated at the 3h and 8h time points. Since AGO1 was a key regulator for miRNAs biogenesis, the down-regulated of AGO1 expression under salinity stress might induce further changes of numerous miRNA activities. This might explain why there were more salinity responsive miRNAs at the 3h and 8h time points than at the 27h (Table 2).

**Fig 8:**
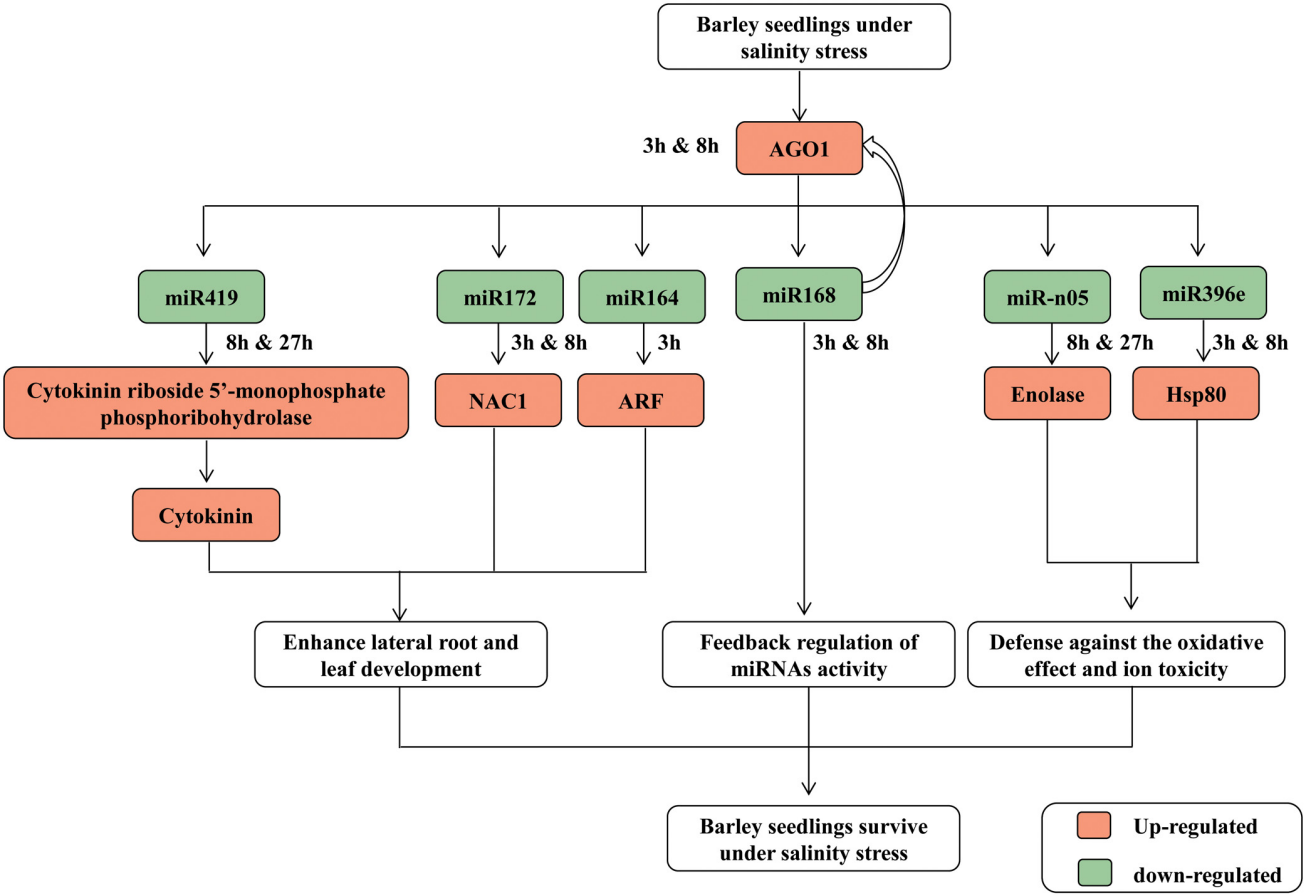
The potential regulatory network of salinity-responsive miRNAs for barley seedling in the early hours of salinity stress. Note: the data contributing to the network were collected from 3h, 8h and 27h salinity stress (100 mm/L NaCl) in barley seedling, and show the potential roles of the salinity responsive miRNAs inbarley.

Here a model has been proposed for the role of miRNAs in the salinity responses of barley seedling under a lower salinity stress. Fig 8 showed that the salinity responsive miRNAs were involved at post-transcriptional levels in the regulation of the metabolic and morphological processes of barley seedling under salinity stress.

### Comparative analysis of salinity responsive miRNAs in plants

Currently, a large number of studies have been performed to reveal the biological mechanism and process of miRNA-mediated salinity stress response in plants, meanwhile an increasing number of salinity-responsive miRNA have been identified [5–8, 10, 53, 55]. In this study, 34 miRNA families were found to be salt-responsive in barely. Compared to other plants, 14 of these miRNA families were also found to be salinity responsive in other plant species. Among them, miR159, miR164, miR167 miR168, miR171, miR172 and miR393 have been demonstrated to be involved in signal transduction by targeting MYB transcription factor, F-box and Nodulation signaling pathway 2 proteins. In addition, three miRNAs including miR319, miR393 and miR396 were targeting genes involved in plant morphological modulation such as plants leaf and root development (S9 Table). Morphological modulation and signal transduction were common survival strategies for plants to adjust to stress [43, 56]. Thus, plants might evolve a conserved miRNA-mediated regulatory mechanism in morphological modulation and signal transduction to response to salinity stress.

Although the majority of the salinity responsive miRNA existed in various plants, the expression patterns of some miRNA were different among species. For example, miR396 was up-regulated expressed in *Arabidopsis* [5], but down-regulated in rice and cotton [52, 53]. Similarly, miR167 was down-regulated in maize [52], but up-regulated in *Arabidopsis* in response to salinity stress [5]. The reasons for these conflicting results might be attributed to the differential experimental conditions or the species specific regulatory networks under salinity stress. These conflicting results highlight the need for more in-depth and detailed characterizations of salinity responsive miRNAs in plants.

Furthermore, some specific salinity responsive miRNA families were also identified. Four miRNAs (namely miR818, miR5071, miR5072 and miR5200), were only found in switchgrass (*Panicum virgatum*) and barley, indicating that these miRNA families might be grass-specific salinity responsive miRNAs. Additionally, 11 miRNA families were newly identified salt responsive miRNA in barley in this study, which could be considered as the barely-specific salt-responsive miRNAs. Their corresponding target genes enriched in the functions of transport, cell wall biogenesis, signal transduction, transcription regulation and other abiotic stress (S9 Table). At the same time, some of them have been reported to display differential expression under other stresses. For example, miR444 has been found to be significantly down-regulated under drought stress in barley [18]. These newly identified salt-responsive miRNAs in barley shed light on the molecular mechanism of barley salt resistance, and also provide invaluable resource to uncover the elite miRNAs for salt resistance improvement in barley and other cereals.

## Conclusions

Barley, renowned as the most salt-tolerant cereal, is an important crop worldwide and widely grown in arid and semiarid regions. In this study, a combination of small RNA and mRNA degradome sequencing was performed to identify miRNAs and their targets in barley under salinity stress. A total of 152 miRNAs (142 conserved and 10 novel ones) were identified, of which 44 miRNAs (39 conserved and 5 novel ones) belonging to 39 families were found as salinity responsive. 14 out of the conserved miRNA families were also found to be salinity responsive miRNAs in other plant species, while 4 and 11 salinity responsive miRNA families were only found in grass and barley, respectively. Attenuated plant growth and decreased metabolic rate are common survival strategies employed to divert energy and other resources to cope with stress condition. 86 and 37 target gene families were found to involve in metabolic process and response to stimulus respectively. Overall, there were at least 39 barley miRNA families and 123 corresponding target gene families response to salinity, which may consist to a miRNA regulatory network in barley. These results could facilitate future research to understand the molecular mechanism of salinity response in barley and related cereals to improve their salinity tolerance.

## Supporting Information

S1 Fig Length distribution of miRNAs identified in barley. (TIF)

S2 Fig Known miRNA with miRNA* sequences identified in the present study. (TIF)

S3 Fig Statistics of the degradome sequencing in barley. Note: The genome sequence of cultivermorex and barley_HighConf_genes were downloaded from IBSC (http://www.public.iastate.edu/~imagefpc/IBSC%20Webpage); HVGI (version 12.0) which was downloaded from DFCI Gene Index and barley_HighConf_genes were used as barley gene model for degradome sequence mapping.
(TIF)

S1 Table Information of primer used for quantitative real-time PCR. Note: a universal oligo dT reverse primer from Invitrogen was used for all miRNA qRT-PCRs.
(XLS)

S2 Table Summary of unique reads and read count identified as various classes of small RNAs. (XLSX)

S3 Table Profile of the known microRNAs (miRBase 20.0) identified in this study. (XLSX)

S4 Table 10 new barley miRNAs identified in this study. Note: LM, length of mature miRNA; LP, length of miRNA precursor; MFE, minimum free energy of candidate precursors; MFEI, the minimum free energy index of candidate precursors; mature position, the position of mature miRNA in the hairpin; pre-miRNA location, the position of candidate precursors in Morex genome sequence.
(XLS)

S5 Table List of predicted targets of conserved miRNAs identified in this study. (XLSX)

S6 Table List of predicted targets of novel miRNAs identified in this study. (XLS)

S7 Table Targets of all barley miRNAs identified in this study by degradome sequencing. Note: TP100M: Transcripts per 100 million; P-value < = 0.05 using Cleaveland pipeline
(XLSX)

S8 Table Expression values for miRNAs responding to salinity stress at 3h-, 8h- and 27h time point. (XLSX)

S9 Table miRNAs responsive to salinity stress in diverse plant species. (XLS)
