# Supplementary figures and images for: Global Identification of MicroRNAs and Their Targets in Barley under Salinity Stress
Source: PLoS One. 2015 Sep 15;10(9):e0137990. doi: 10.1371/journal.pone.0137990 (PMC4570814; doi:10.1371/journal.pone.0137990)

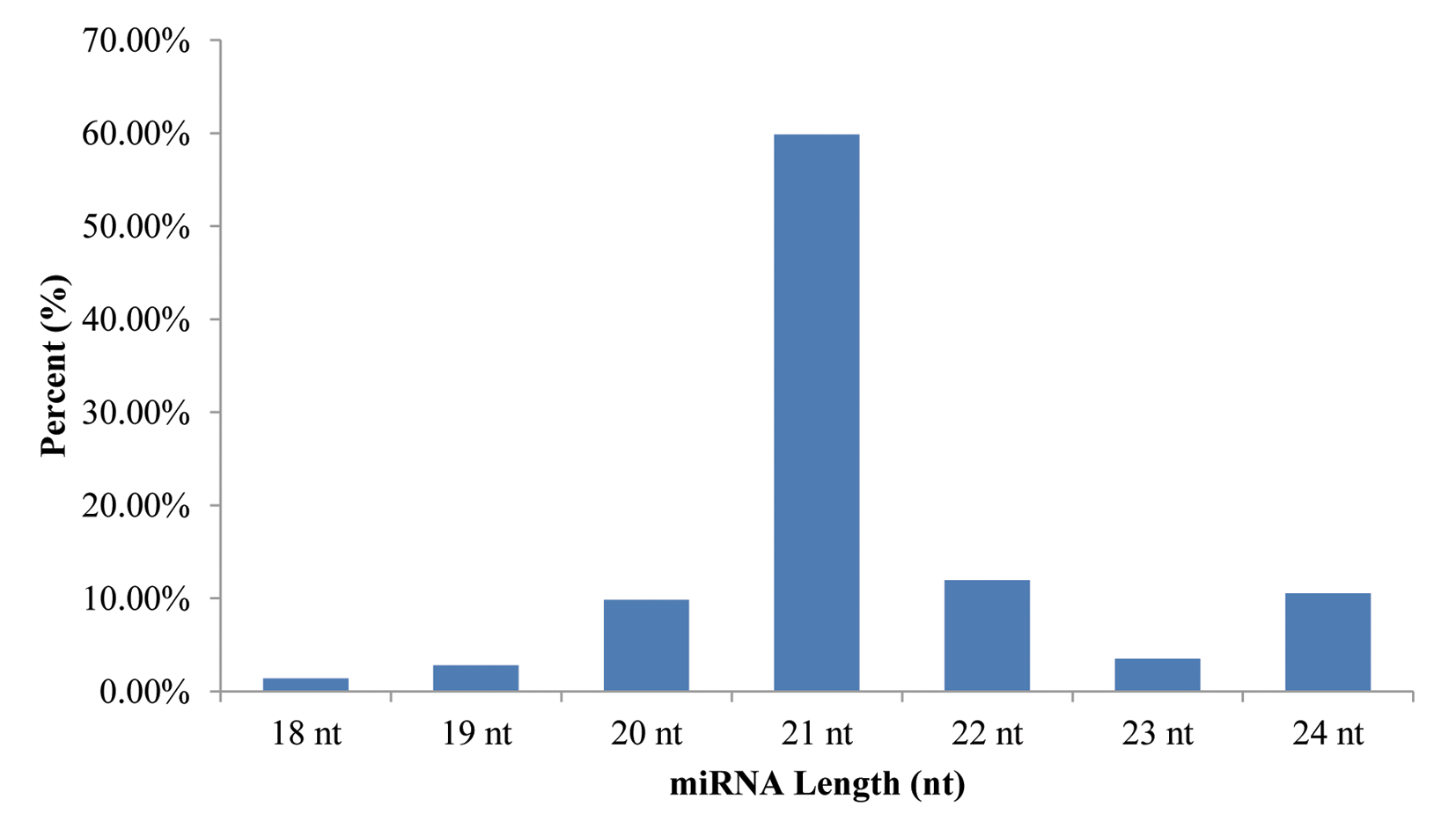

Supplement: S1 Fig — (TIF) [file pone.0137990.s001.tif]

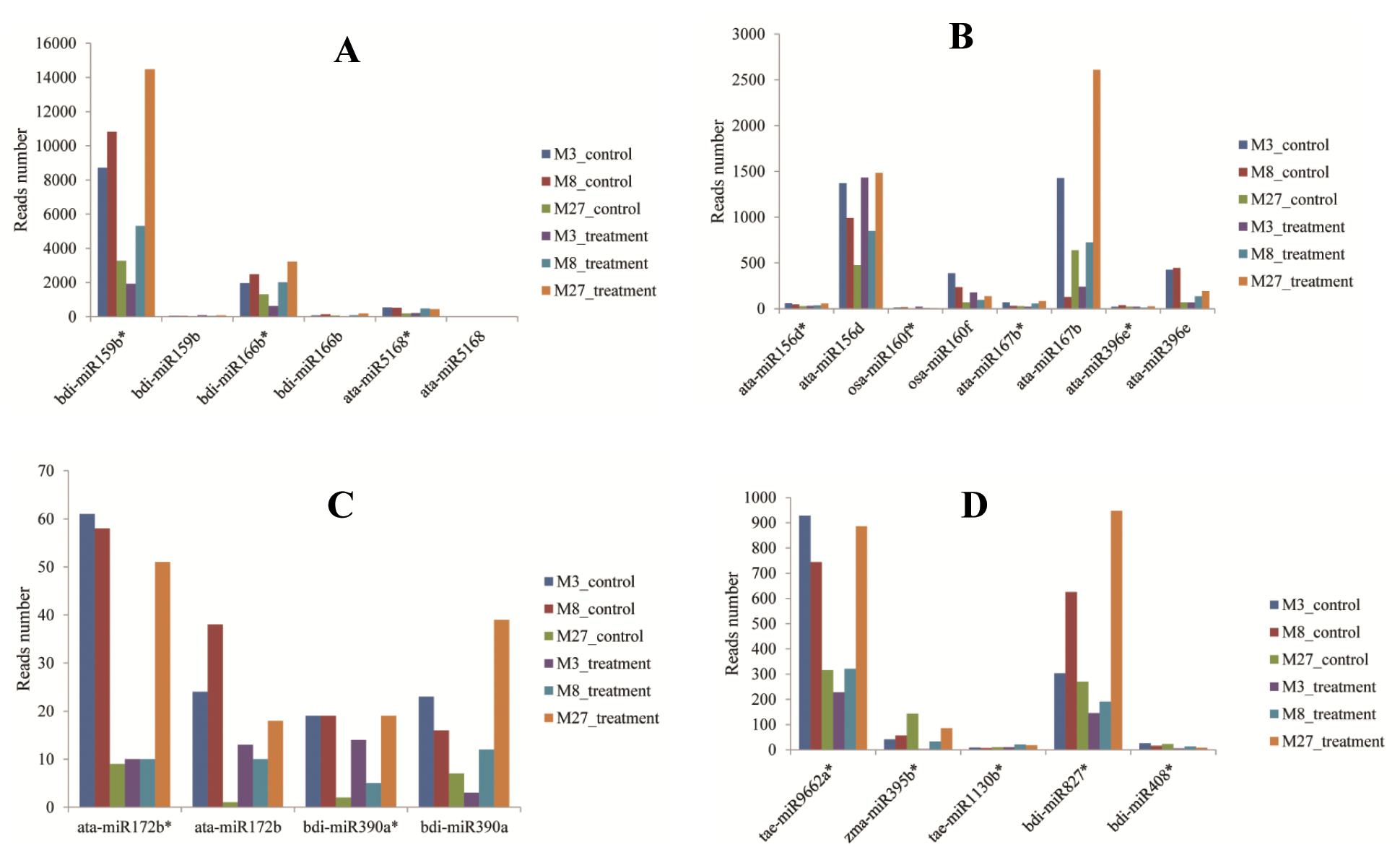

Supplement: S2 Fig — (TIF) [file pone.0137990.s002.tif]

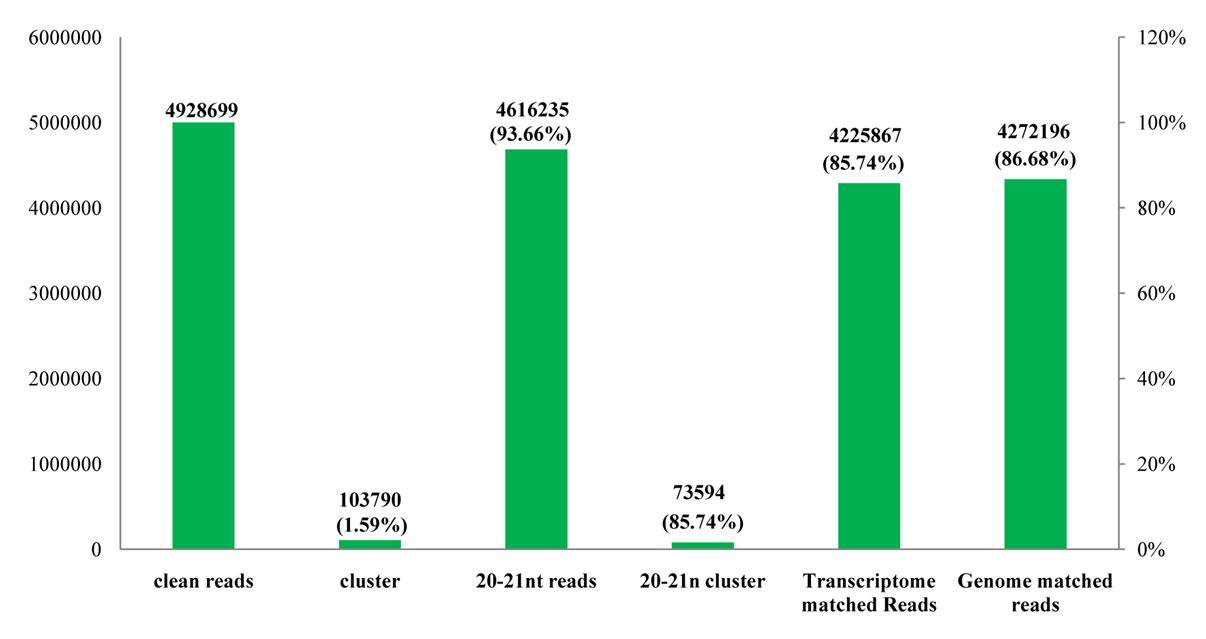

Supplement: S3 Fig — Note: The genome sequence of cultivermorex and barley_HighConf_genes were downloaded from IBSC (http://www.public.iastate.edu/~imagefpc/IBSC%20Webpage); HVGI (version 12.0) which was downloaded from DFCI Gene Index and barley_HighConf_genes were used as barley gene model for degradome sequence mapping. (TIF) [file pone.0137990.s003.tif]
